# Quantification of surface tension and internal pressure generated by single mitotic cells

**DOI:** 10.1038/srep06213

**Published:** 2014-08-29

**Authors:** Elisabeth Fischer-Friedrich, Anthony A. Hyman, Frank Jülicher, Daniel J. Müller, Jonne Helenius

**Affiliations:** 1 Max Planck Institute for the Physics of Complex Systems, Nöthnitzer Strasse 38, 01187 Dresden, Germany; 2 Max Planck Institute of Molecular Cell Biology and Genetics, Pfotenhauerstr. 108, 01307 Dresden, Germany; 3 Department of Biosystems Science and Engineering, Eidgenössische Technische Hochschule Zürich, Mattenstr. 26, 4058 Basel, Switzerland

## Abstract

During mitosis, adherent cells round up, by increasing the tension of the contractile actomyosin cortex while increasing the internal hydrostatic pressure. In the simple scenario of a liquid cell interior, the surface tension is related to the local curvature and the hydrostatic pressure difference by Laplace's law. However, verification of this scenario for cells requires accurate measurements of cell shape. Here, we use wedged micro-cantilevers to uniaxially confine single cells and determine confinement forces while concurrently determining cell shape using confocal microscopy. We fit experimentally measured confined cell shapes to shapes obeying Laplace's law with uniform surface tension and find quantitative agreement. Geometrical parameters derived from fitting the cell shape, and the measured force were used to calculate hydrostatic pressure excess and surface tension of cells. We find that HeLa cells increase their internal hydrostatic pressure excess and surface tension from ≈ 40 Pa and 0.2 mNm^−1^ during interphase to ≈ 400 Pa and 1.6 mNm^−1^ during metaphase. The method introduced provides a means to determine internal pressure excess and surface tension of rounded cells accurately and with minimal cellular perturbation, and should be applicable to characterize the mechanical properties of various cellular systems.

At the entry to mitosis most animal cells change shape to become largely spherical. Cells, both in tissue and when grown in culture, undergo mitotic cell rounding^1^,^2^,^3^,^4^. By rounding, cells gain a defined geometry and sufficient space for a mitotic spindle with proper orientation and correct chromosome segregation^5^,^6^,^7^,^8^. A key player in the determination of cell shape is the actomyosin cortex - a thin actin-rich layer underneath the plasma membrane^9^,^10^,^11^. This cytoplasmic layer consists of a meshwork of polymerized actin and actin-binding proteins. Active myosin motors cross-link cortical actin polymers and exert forces that give rise to active mechanical stress in the cortical layer^9^. This cortical stress together with membrane tension leads to an effective cell surface tension that promotes a reduction of cell surface area^11^.

At the entry to mitosis, the actin cytoskeleton undergoes a drastic reorganization directed by the mitotic CylinB-Cdk1 complex^12^; F-actin is enriched at the cell periphery and myosin II gets activated, regulated by the Cdk1 substrate Ect2 and its downstream effector RhoA^13^,^14^,^15^. This actin reorganization is essential for increased cell surface tension and cell-rounding in mitosis^14^,^16^. Measuring the force exerted by confined mitotic HeLa cells, Stewart *et al.* inferred that the increasing contractile stress in the cell cortex is balanced by an increasing internal hydrostatic pressure^17^. This conclusion was based on cells modeled as pressurized liquid sacks bounded by a shell in which contractile in-plane tensions are present. The cell boundary is then governed by Laplace's law which relates internal pressure excess, tension and curvature (see Supplementary Section 1 online). Stewart *et al.* chemically perturbed different cellular systems including F-actin, microtubules and ion homeostasis and found effects consistent with Laplace's law. However, whether the shapes of confined cells obey Laplace's law has not been examined and the cell surface tension of the HeLa cells was only coarsely estimated.

Here, we examine rounded interphase and mitosis HeLa cells uniaxially confined between a wedged micro-cantilever and a coverslip^18^. Simultaneous confocal imaging of cells with fluorescently labeled cortex allows the cell boundary and, thus, the cell shape to be determined while the confinement force is measured. We consider cells as a liquid core surrounded by a thin cortical shell (≈ 200 nm in thickness^28^) that is under mechanical tension^11^,^19^,^20^. Cell shapes are then calculated using Laplace's law^21^,^22^ and fit to measured cell shapes. The thereby obtained accurate geometrical parameters of cell shape are used to calculate the internal hydrostatic pressure excess and the surface tension of the cell from the confinement force exerted by the micro-cantilever on the cell. We measure pressure excess and surface tensions of cells undergoing mitosis and compare these values with those obtained for non-adherent interphase cells.

## Results

### Shapes of confined cells

**Figure 1:**
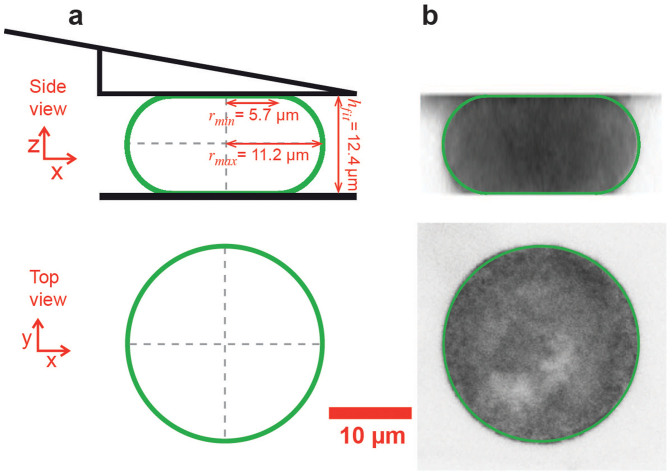
Parallel plate confinement of rounded HeLa cell. (a) Sketch of the theoretically predicted cell surface (green). Shown are the dimensions of the minimal cross-sectional radius (*r_min_*), the equatorial radius (*r_max_*) and the height (*h_fit_*). This shape is the fit theoretical shape associated to the cell depicted in B. (b) 2-D renderings of a confocal z-stack taken from a confined hMYH9-LAP expressing HeLa cell in metaphase. Overlaid in green is the fit theoretical shape. The rendered cell shape seems to deviate from the fit theoretical shape at the top and the bottom. This effect can be accounted for by the combination of limited z-resolution and the cell surface being almost parallel to the image plane. The z-interval was 0.5 *µ*m and the pixel size was 76 nm.

**Figure 2:**
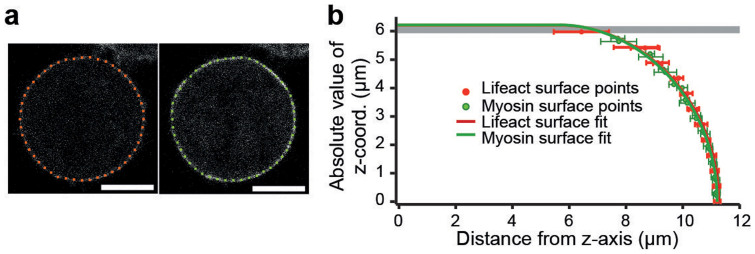
Applying the cortical shell-liquid core model to a confined cell. (a) Confocal images of the cell shown in Fig. 1. The two images are Lifeact-mCherry (red) and hMYH9-LAP (green) channels of the same cell and z-plane near mid height. Red and green points denote the shape of the cell boundary as determined by cell edge detection. Scale bars, 10 *µ*m. Fluorescence intensities of cell boundary, cytoplasm and background relate typically as 300/60/6. (b) Comparison of measured cell shape and fit theoretical shape. Solid red and green lines represent the vertical cross section of the rotationally symmetric profile of the fit shape. Data points show mean and standard deviation of surface points representing the measured cell shape in one z-plane (red: Lifeact-mCherry, green: hMYH9-LAP).

We performed a parallel plate confinement assay on HeLa cells using a combined confocal microscopy and AFM setup (Fig. 1). Measured cells were either in mitosis or not adherent and, therefore, largely spherical prior to confinement with the cantilever. Cells either expressed two fluorescent actomyosin cortex labels (hMYH9-LAP and Lifeact-mCherry) or mCherry-CAAX which predominantly locates to the plasma membrane. To find the shape of confined cells confocal z-stacks were recorded and analyzed. In each image of a stack, the cell borderline was determined as described in the Supplementary Section 6 online. 48 discrete equidistant points represent the cell border in each image (Fig. 2a). The points of all z-stack images recorded within the cell were combined and represent the three-dimensional surface of the cell. The closest theoretical shape, parameterized by its center point and two cross-sectional radii (*r_min_* and *r_max_*), was determined by a fit. Geometric parameters obtained by fitting the surface points of seven metaphase cells are summarized in Supplementary Table S1 online. An analogous analysis for interphase cells is shown in Supplementary Table S2 online. The average distance < *d* > between measured surface points and the fit surface is smaller than 300 nm for all fits, demonstrating the good agreement between the measured cell shape and the cell shape predicted by the model (Fig. 2b).

### Uncertainty of cell geometry determination

**Figure 3:**
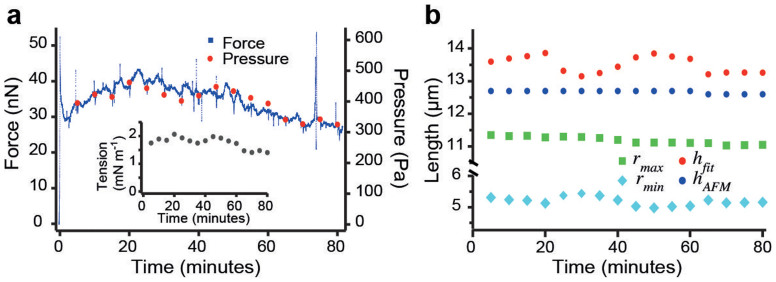
Reproducibility and uncertainty of experimental results. (a) Force (small blue dotes) and hydrostatic pressure difference (red dots) of an mCherry-CAAX expressing HeLa cell arrested in mitosis over time. The inset shows the associated surface tension. (b) Physical dimensions, *r_max_*, *r_min_* and *h_fit_* as determined by a fit of theoretical cell shapes and the cell height as measured by the AFM, *h_AFM_*, at the given times.

Fitting cell shapes allows us to accurately determine geometrical parameters of the shape such as surface curvature and contact areas with the plates. To assess the temporal variability of cell shape and cell mechanics, and to estimate the uncertainty of our fits, the change of cell shape was examined over time. For this purpose, an mCherry-CAAX expressing HeLa cell was arrested in mitosis by the addition of S-trityl-l-cysteine (STC) to the media^23^. STC prevents mitotic cells from entering anaphase for extended time periods by inhibiting Eg5, a mitotic spindle kinesin. We confined the cells for 80 minutes and observed that the force exerted on the cantilever remained within a 30% range of the mean (Fig. 3a). Confocal z-stacks were recorded at five-minute intervals and cell shape parameters were determined from each z-stack. The hydrostatic pressure excess was calculated using the force at the time the stack was recorded (Fig. 3a). Geometrical parameters of the cell shape are plotted as a function of time in Fig. 3b. While the horizontal radius of the cell remained largely constant, estimated height *h_fit_* and minimal radius *r_min_* varied. Since the cantilever maintained the height of the cell and assuming the shape of the cell was constant, the variations in geometrical parameters represent the uncertainty of the measurements. The standard deviation of the measured minimal radius *r_min_* is 140 nm, which corresponds to 2.6%. Measurements of the equatorial radius *r_max_* are more accurate with a standard deviation of only 1%. Using error propagation, the uncertainty in the minimal cross-sectional radius suggests a relative error of the pressure of 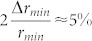. This compares to a relative standard deviation of the measured force (and therefore of the actual pressure excess if the cell shape remains unchanged) of ≈ 12% in the time interval of observation.

### Cell surface tension is independent of the degree of uniaxial cell confinement

We examined STC arrested cells (*n* = 5) confined to several heights (≥4, between 10 and 40% reduction of unconfined cell height) and calculated pressure excess and surface tension (see Supplementary Fig. S4 online). At limited confinement (< 20%), radii of the cell-plate contacts are small. Therefore, the relative error of the contact area becomes large, which implies a large error in pressure and tension values. We did not allow the reduction of cell height to exceed 50% because the cells usually extrude large blebs (> 5 *µ*m long) if their height is reduced further. We find that the cell surface tension stayed largely constant within ±10% independent of the degree of confinement. The averaged (normalized) hydrostatic pressure excess increased slightly with the degree of cell confinement. This rise is expected due to the increase in the mean curvature of the cell surface at lower cell heights, as pressure excess is the product of tension and twice the mean curvature (Eq. 1). We conclude that the degree of confinement of a cell within the range used in our experiments does not crucially affect the cell's surface tension in mitosis.

### Surface tension and cell shape

**Figure 4:**
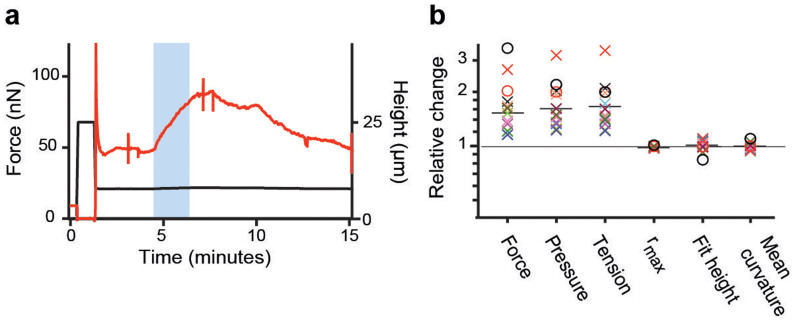
Tension dependence of cell shape in metaphase. (a) Confinement force (red) recorded over time while a parallel plate confinement assay was performed on a HeLa cell in the presence of 10 *µ*M blebbistatin. The blue region indicates when the cell was illuminated with blue light to photo-inactivate blebbistatin. Spikes in the force trace at 3 and 8 minutes indicate the recording of mCherry confocal z-stacks. The confinement height (black) is plotted below. (b) Normalized photo-inactivation induced changes in the properties of blebbistatin treated HeLa cells in metaphase (*n* = 13). Crosses indicate experimental measurements whereas circles indicate relative changes predicted by theory for the case of finite contact angle (black circle: *φ* = 45° → 31.4°, red circle: *φ* = 10° → 7.06°). Black bars indicate the mean of experimental data points. Changes in contact angle due to tension rise were calculated according to Eq. 2. Surface tension *γ* was anticipated to rise from 0.6 to 1.2mNm^−1^ with a cell volume of 4800 *µ*m^3^ and a cantilever height *h_AFM_* of 12 *µ*m.

Provided the contact angle of the cell is zero, analogous to the case of a perfectly dewetting droplet, and anticipating that cell volume is constant, our model implies that the shape of a cell confined between parallel plates is independent of surface tension and pressure excess as long as they are high enough to dominate over effects of adhesion and gravity. Conversely, the confinement force depends on surface tension and pressure excess. To test if this applies to confined HeLa cells, we used the myosin II inhibitor blebbistatin^24^, which is photo-inactivatable^25^. HeLa cells in metaphase were confined in the presence of blebbistatin and a confocal z-stack was recorded. In this state, surface tension of cells was significantly smaller than average metaphase values but still ≥ 0.4 mNm^−1^. Thereafter, the blebbistatin in the cell was deactivated by wide-field blue light excitation and a second z-stack was acquired. Fig. 4a renders the process. Upon photo-inactivation, the force exerted by the cell on the cantilever increased as myosin II was no longer inhibited. As active blebbistatin re-entered the cell from the medium, and again inhibited myosin II, the exerted force returned to the lower starting value. Comparing measured cell parameters, we find that cortex tension and pressure excess increase substantially due to photo-inactivation of blebbistatin, whereas the parameters describing the cell shape do not change significantly (Fig. 4b, Table 1). Since myosin II increases the contractility of polymerized actin meshwork^26^, this data supports the applicability of the cortical shell-liquid core model and that myosin II actively maintains the high tension of the cortex^14^,^27^. Furthermore, the fact that cell shape parameters depend weakly on surface tension indicates that contact angles are small or vanishing.

### Hydrostatic pressure excess and tension increase during mitosis

**Figure 5:**
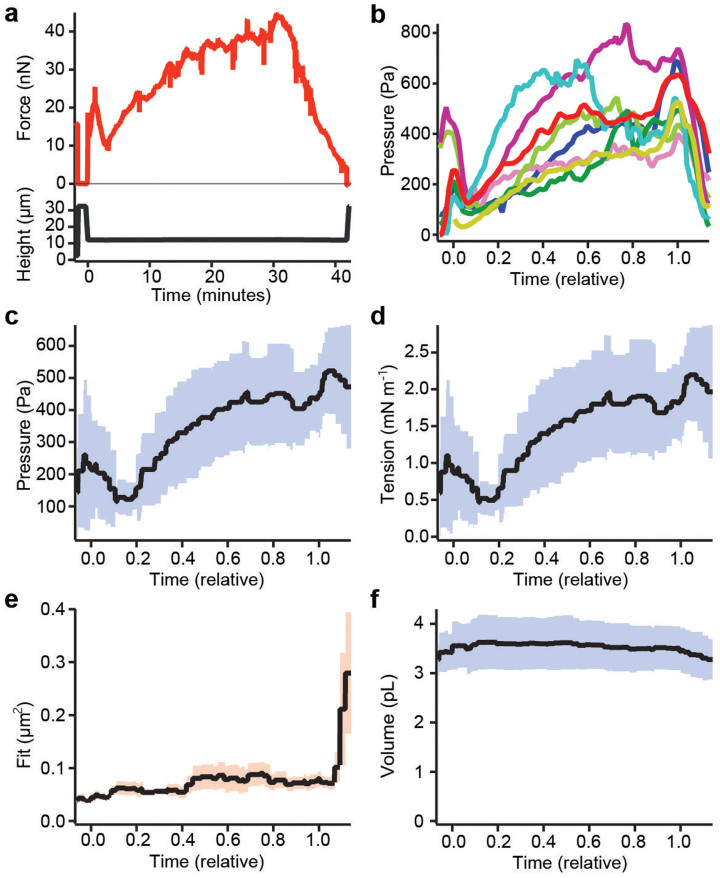
Cell properties during mitosis. (a) Confinement force (red) recorded over time from a parallel plate confinement assay performed while a pre-rounded HeLa cell completed mitosis. Spikes in the force trace at 5-minute intervals indicate the recording of confocal z-stacks. The confinement height (black) is plotted below. (b–f) HeLa cells (*n* = 8) measured during the course of mitosis. Plots from different cells were aligned in a relative time scale such that time zero coincides with the moment of nuclear envelope breakdown and time 1 with the start of chromosome separation in anaphase. (b) Hydrostatic pressure difference over relative time during mitosis. (c) Mean pressure of cells. The blue shaded region represents the standard deviation. (d) Mean surface tension. (e) Mean squared fitting error for the confocal z-stacks over relative time. The orange shaded region represents the standard error of the mean. (f) Mean volume of the mitotic cells over relative time.

Having confirmed our model of cell shape, we determined how the hydrostatic pressure excess and surface tension of the cell change while the cell undergoes mitosis. As HeLa cells are normally spread on a substrate and not spherical in prophase at the beginning of mitosis, we de-adhered cells using trypsin (Methods). Non-adherent cells are spherical, because this is the shape with minimal surface favored by surface tension. We applied parallel plate confinement at constant height to cells in prophase or early prometaphase as judged by chromosome condensation. As cells proceeded through mitosis, the AFM force was recorded continuously while confocal z-stacks were recorded at five-minute intervals (Fig. 5a). Shape parameters were determined by a fit. Together with the force data, they were used to calculate cortex tension and pressure excess throughout mitosis until anaphase when cleavage furrow ingression has distorted the shape of the cell. While early mitotic cells had low surface tension and pressure excess, both quantities exhibited a small, sharp peak (< 5 minutes) at nuclear envelope breakdown (Fig. 5b). After this peak, both tension and pressure excess increased further during prometa-and into metaphase reaching on average values of 1.6 mN m^−1^ and 400 Pa in metaphase (Fig. 5c and d). Assuming a cortical thickness of ≈ 200 nm^28^, this surfaces tension implies cortical stresses of ≈ 8 kPa in metaphase. In anaphase, an isolated cell becomes non-spherical suggesting non-uniform cortical tension and thus our model can no longer be used. A decrease in the quality of the fit is observed at this time (Fig. 5e). Although the pressure excess increased more than threefold during mitosis the volume of the cell, in agreement with^17^, remained largely constant within the uncertainties of our measurement (≈ 10%, Fig. 5f).

**Figure 6:**
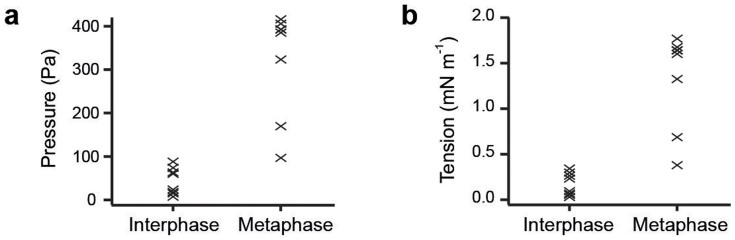
Comparison of hydrostatic pressure difference and surface tension of interphase versus metaphase cells. Plotted are the hydrostatic pressure excess (a) and surface tension (b) of non-adherent interphase and mCherry-CAAX expressing HeLa cells in metaphase (*n* = 8) confined to a height of ≈ 12 *µ*m between an AFM cantilever and a PDMS surface. Pressure excess and tension were calculated using confocal stacks with a z-interval of 0.5 *µ*m. Cell phase was determined using H2B-eGFP confocal images.

To compare pressure excess and tension values of mitotic cells to those of interphase cells, we performed measurements on un-synchronized trypsin-rounded cells. The cell cycle state of each cell was judged based on H2B-eGFP morphology. We found average hydrostatic pressure differences of 40 ± 30 and 320 ± 120 Pa and surface tensions of 0.17 ± 0.13 and 1.3 ± 0.5 mN m^−1^ for interphase (*n* = 8) and mitotic (*n* = 8) cells, respectively (Fig. 6).

### Pressure and tension measurements using single z-plane imaging

If the contact angle of the cell with the confining plates is zero, the cell shape is entirely determined by the cell's height and its radius at the equator. These two parameters can be determined using cantilever height and a single fluorescence or DIC micrograph of the equatorial plane of the cell. Since our estimation of contact angles indicates small values ≤ 10° the assumption of zero contact angles can be regarded as an acceptable approximation. The use of single plane imaging results in qualitatively similar results but with increased uncertainties (≤ 10% error, see Supplementary Table S2 online). This simplified method circumvents photo-damage of the cell and extensive experimental and analytical effort.

## Discussion

We introduce a method to measure the surface tension and hydrostatic pressure excess of non-adherent HeLa cells, using AFM-based parallel plate confinement. The method relies on the cortical shell-liquid core model that stipulates a uniform hydrostatic pressure inside the cell and homogeneous cell surface tension. The model specifies the rotationally symmetric shape and the mechanics of non-adherent cells confined between parallel plates (Fig. 1 and 2).

Parallel plate assays are an established method to probe mechanical properties of cells^21^,^29^,^30^,^31^,^32^,^33^. Measurements of cell surface tension, typically using micromanipulators, were performed on large cells (> 50 *µ*m in diameter) such as sea urchin eggs^21^,^29^ and plant cells^30^. Use of AFM permits parallel plate confinement of cultured animal cells (often < 20 *µ*m in diameter)^18^.

We performed confocal imaging to locate the surface of confined cells using three different fluorescent constructs (mCherry-CAAX, Lifeact-mCherry, hMYH9-LAP). In general, we find good agreement between fit cell shapes and measured cell surface points (see Supplementary Table S1 and S2 online). This is demonstrated by deviations of less than 300 nm between measured and theoretical cell shape for metaphase and interphase cells imaged with z-intervals of Δ*z* = 0.5 *µ*m. Analysis of confocal z-stacks did not show qualitative differences between the different fluorophores. In fact, cells that co-expressed Lifeact-mCherry and hMYH9-LAP were imaged in two fluorescence channels simultaneously and the obtained cell geometries coincide within the error of the measurements (see Supplementary Table S1 online).

If we compare the height of the fit theoretical shape with the actual height of a cell, which is set by the AFM cantilever, we find good agreement. On average, the height of the fit shape exceeds the actual cell height by ≈ 5% in metaphase and ≈ 4% in interphase, which corresponds to 400–600 nm. This discrepancy could originate from the presence of small but non-vanishing contact angles (see Supplementary Fig. S2 online) or from experimental uncertainties. The observed height differences are smaller than the typical error of the fit height (≈ 1 *µ*m, see Supplementary Section 2 online). To further test for the influence of cell adhesion, we examined the extent to which cells can exert downward forces on the cantilever. Theory predicts that in the case of non-vanishing adhesion, a cell would pull the cantilever downward when the distance between the parallel plates is slightly smaller or greater than the height of the round cell (see Supplementary Fig. S3 online). The expected downward force increases with cell surface tension and contact angle. To probe this experimentally, we first squeezed mitotic cells and then slowly lifted the cantilever until it lost contact with the cell. We find that the cells exert little or no downward forces (*F_AFM_* ≥ −1 nN) on the cantilever (see Supplementary Fig. S3a online). Comparing the measured forces to theoretical predictions, we conclude that the contact angle of a metaphase cell is ≤ 10°.

A direct consequence of weak adhesion and the negligible contact angle at the plates is, that the cell shape is independent of the actual value of cell surface tension as long as surface tension suffices to dominate over other cell shaping effects such as adhesion, gravity and internal cytoskeletal structures (Eq. 2). This prediction of theory was confirmed by measurements of blebbistatin treated cells in metaphase. Blebbistatin inhibits myosin II and, thus, reduces the contractility of the actomyosin cortex and the surface tension of the cell. Blue light inactivates blebbistatin and transiently increases the surface tension of blebbistatin treated cells. As predicted by theory, the shape of cells did not change within the uncertainties of our measurement upon photo-inactivation of blebbistatin (Fig. 4).

We find that both surface tension and hydrostatic pressure change during the cell cycle of HeLa cells. Interphase cells had a consistently lower hydrostatic pressure excess and surface tension (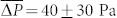 and 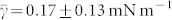, *n* = 8, Fig. 6). During mitosis, pressure excess and tension increased, assuming peak values of 400 ± 120 Pa and 1.6 ± 0.5 mN m^−1^ during metaphase (Fig. 5 and 6). Therefore, HeLa cells enhance their internal pressure excess and surface tension by one order of magnitude when they enter mitosis. Thereby, cells contribute to achieving a spherical cell shape that allows unperturbed assembly of the mitotic spindle. In fact, if we incubate adhered cells with blebbistatin on uncoated glass bottom dishes, we often observe that cells do not round up entirely until metaphase in contrast to untreated cells (see Supplementary Fig. S7 online). Cell surface tension in mitosis appears to be mainly generated by the actomyosin cortex because surface tension can be reduced to values ≤ 10% of that of untreated cells by inhibition of myosin II together with actin-depolymerizing drugs such as latranculin A or cytochalasin D. We estimate that the contribution of membrane tension of mitotic Hela cells to overall cell surface tension is ≤ 0.2 mN m^−1^, which is in accordance with previous measurements of cell membrane tension^34^. Therefore, we conclude that the change in surface tension is mainly generated by changes in the cortical properties of the cell at the onset of mitosis^17^. This includes the activation of myosin II^14^.

The surface tension of cells can also be measured using other methods, such as micropipette aspiration^35^,^36^. The surface tensions that we measure for HeLa cells in interphase are on the same order of magnitude as surface tensions of fibroblasts measured by micropipette aspiration^9^. A study of mitotic HeLa cells using micropipette aspiration is unknown to us. We speculate that micropipette suction may interfere with mitotic progression due to deformation of the mitotic spindle and could trigger mechanosensitive responses^7^,^37^. HeLa cells confined in our assay complete mitosis in the same time as unconfined cells unless confined to less than 40% of their unconfined height. An estimate of surface tension in mitotic HeLa cells, that is in agreement with our work, was obtained by Charras *et al.* from studies of the volume of blebs. Their estimate is 0.13–0.9 mN m^−1^ 38.

How do surface tension and pressure relate to each other? As the cell up-regulates its surface tension in mitosis, the internal hydrostatic pressure rises immediately according to the Laplace law. A simple physical argument can explain why the osmotic pressure inside the cell adjusts in response to the tension increase. At steady state, the hydrostatic pressure difference across the cell boundary is balanced by the difference of osmotic pressures across the cell boundary. This follows from the equality of chemical potentials of water inside and outside of the cell^39^. Because of the hydrostatic pressure increase, the chemical potential of water in the cell exceeds the chemical potential in the medium. Therefore, water flows out of the cell increasing the osmolyte concentration in the cytoplasm and, thereby, the osmotic pressure. Water outflux continues until the osmotic pressure difference equals the raised hydrostatic excess difference in the cell. In this way, the osmolarity of the cell can be increased by water outflux that does not involve the action of ion channels or pumps. However, active ion pumps are needed to maintain the osmolarity gradient across the cell membrane, otherwise, cells cannot reach a steady volume at non-vanishing surface tension.

The duration of water fluxes after a surface tension change can be estimated to lie between 1–5 min, assuming water permeabilities of our cells between 0.001 and 0.005 cm s^−1^ 40. Active cell volume regulation operates on slightly slower time scales^41^. Contrary to Boucrot *et al.*^42^, we did not observe a substantial change in the cell volume during mitosis, which is in agreement with earlier findings^17^. While mitotic volume changes clearly need to be more thoroughly studied to resolve this issue, our use and verification of cortical-shell liquid-core model does not depend on the cell volume remaining constant during mitosis.

Using Π = *RTc*, where Π denotes osmotic pressure and *c* osmolarity, we find that the osmolarity difference Δ*c* between the cytoplasm of an interphase cell and the medium is ≈ 0.016 mOsm in steady state. For mitotic cells, we obtain Δ*c* ≈ 0.13 mOsm. These osmolarity differences are small compared to the absolute osmolarity of the medium (≈ 300 mOsm).

It is tempting to speculate on the cause of the peak in hydrostatic pressure excess and surface tension during nuclear envelope breakdown (Fig. 5). Osmolytes within the nucleus might be released into the cytosol increasing its osmolarity giving rise to a slight osmotic swelling of the cell. The accompanying cortical stretching could give rise to a temporary elastic increase of the surface tension. Over time, this tension increase relaxes due to the turnover of actin and/or actin cross linkers^9^. An alternative hypothesis is that factors, such as Cdk1 and Ect2^15^,^43^, are released during nuclear envelope breakdown, which cause the cortex to transiently contract and, thereby, temporarily the pressure excess to increase.

Our results show that a simple cortical shell-liquid core model of the cell describes the shape of non-adherent stationary HeLa cells. It can be used to quantify physical properties such as hydrostatic pressure excess and surface tension. We expect that this method can be applied to other cell types and yield insight into fundamental cell biological processes, such as osmoregulation and cortex dynamics.

## Methods

### Force balances that determine cell shape

Consider a cell that is confined between two parallel plates. The coarse-grained cell shape is defined by a surface that we refer to as cell surface. It consists of a curved part in contact with the medium and two at areas in contact with the confining plates (Fig. 1a). The cell is bound by a thin cortical shell, which consists of a meshwork of polymerized cross-linked actin and the attached cell membrane. Myosin motors bind to cortical actin polymers and exert forces that give rise to active mechanical stresses leading to an effective cell surface tension *γ*^9^,^27^. The cell interior, which we regard as a liquid, is characterized by an internal hydrostatic pressure. If the bending energy of the shell is negligible, the force balance at the cell surface in contact with the medium is described by the Laplace equation^21^,^44^ (see Supplementary Section 1 online) 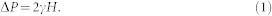
Here, Δ*P* is the hydrostatic pressure difference between cell interior and medium, and *H* is the mean curvature at a point on the cell surface.

Attractive interactions between the cell surface and the confining plates can be characterized by an energy per unit area *W*. The contact angle *φ* of the cell surface with the confining plates is determined by the force balance at the contact line. According to the Young-Dupré equation, the contact angle fulfills the relation^44^,^45^

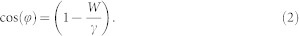
In the following, we consider only cells that were either de-adhered or in mitosis. We observe that such cells are to a large extent rotationally symmetric in the confinement assay. Rotationally symmetric shapes, as predicted by the theory, can be calculated for a given cell volume using Eqs. 1 and 2 (Supplementary Section 1 online). These theoretically predicted shapes are surfaces of revolution generated by a curve *r*(*z*), where *r*(*z*) is the shape's cross-sectional radius along the z-axis. The associated tangential angle is denoted as *θ*(*z*) with tan(*θ*(*z*)) = −*dz*/*dr* (see Supplementary Fig. S2 online). In the chosen coordinate system, the cross-sectional radius *r*(*z*) reaches its summit at *z* = 0, giving *θ*(0) = *π*/2. We refer to the x-y cross-section at *z* = 0 as the shape's equator.

In our cell confinement assay, cells are confined between the bottom of the cell culture dish and an AFM cantilever supplemented with a solid wedge (Fig.1a)^18^. We refer to the lowermost layer of the wedge as the top confining plate. In order to relate the cell shape to the force exerted by the cantilever *F_AFM_*, consider the force balance condition at the interface of the cell with the top confining plate. The cantilever force is balanced by the force due to the hydrostatic pressure excess inside the cell and the z-component of the force associated with the cell surface tension^44^, i. e. 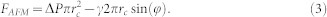
Here, *r_c_* is the radius of the contact area of the cell with the confining plate. This argument can be generalized. To any x-y cross section through the cell, we can associate a vertical force resulting from the integrated hydrostatic pressure excess and from the surface tension 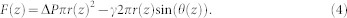
Force balance requires that this force is independent of *z* and, therefore, *F_AFM_* = *F*(*z*). For the particular case of *z* = 0, the x-y extension of the shape is maximal and *θ* = *π*/2 (see Supplementary Fig. S2 online). Therefore, we have 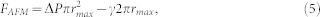
where *r_max_* is the cross-sectional radius at *z* = 0. If the contact angle *φ* is non-zero, it is possible to extend the mathematical surface describing the cell shape beyond the confining plates until a cross section with tangential angle *θ*(*z*) = 0 is reached using the Laplace law (see Supplementary Fig. S2b online). The radius *r_min_* of the outermost cross section of this extended shape thus fulfills the relation 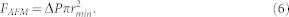
The height of this extended shape is denoted as *h_fit_*. If the contact angle *φ* is zero, then the contact radius of the calculated cell shape equals *r_min_* and the cell height *h_AFM_* equals *h_fit_*. For finite contact angle, the contact radius of the calculated shape *r_c_* > *r_min_* and *h_AFM_* < *h_fit_*.

### Calculating cell surface tension and cell pressure excess from experimental data

The fit theoretical shape is parameterized by its two cross-sectional radii *r_min_* and *r_max_*. Using *r_min_* as determined from the cell shape and *F_AFM_*, we calculate the pressure excess as 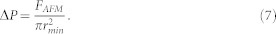
Similarly, the surface tension is obtained as 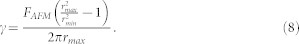
This relation follows from Eqs. 1, 5 and 6.

### Cell culture

Two HeLa-Kyoto cell lines expressing either histone H2B-eGFP (H2B-eGFP) and mCherry-CAAX^17^ or Lifeact-mCherry and hMYH9-LAP were maintained in DMEM supplemented with 10% fetal bovine serum, 2 mM Gluta-MAX, 100 µg ml^−1^ penicillin, 100 µg ml^−1^ streptomycin, 0.5 µg ml^−1^ puromycin and 0.5 mg ml^−1^ geneticin (all Invitrogen) at 37°C with 5% CO_2_. For experiments the media was changed to DMEM with 4 mM Na_2_CO_3_ buffered with 20 mM HEPES/NaOH pH 7.2. Where noted, blebbistatin(-) (Chemie GmbH) or S-trityl-l-cysteine (STC, Sigma) dissolved in DMSO were added to concentrations of 10 or 2 µM, respectively, before the experiment.

### Instrumentation

The experimental set-up consisted of an AFM (Cellhesion 200, JPK Instruments) mounted on an inverted confocal microscope (Observer.Z1, LSM 700, Zeiss). A 63x/1.3 LCI Plan-Neofluar water immersion objective (Zeiss) was used. The cells were maintained at 37°C using a Petri dish heater (JPK Instruments). 350 m long, nominal 150 mN m^−1^ cantilevers (NSC12/tipless/noAl, Mikromasch) were modified with epoxy (ET302, Epoxy Technology) wedges to correct tilting and thus allow axisymmetric confinement^18^. Unless otherwise noted, images in confocal z-stacks were recorded 0.5 *µ*m apart with pixed size of 76 × 76 nm^2^.

### Parallel plate confinement assay

For parallel plate confinement assays, cells were kept in CO_2_ independent media at 37°C on glass bottom Petri dishes (FD35, WPI). First, a cell at the desired stage of its cell cycle was identified by its shape and/or H2B-eGFP. Then, the AFM cantilever was lowered to the dish near the cell until it came into contact with the surface. The cantilever was raised (usually 25 *µ*m), positioned above the cell and lowered at 1 *µ*m s^−1^ to confine the cell to a predetermined height. The height of the cantilever was maintained at this height for the duration of the experiments and while fluorescent images were recorded. The force acting on the cantilever was continuously recorded. The height of the confined cell can be computed as the difference between the height that the cantilever was raised from the dish surface and lowered onto the cell plus the height of spikes at the rim of the wedge (due to imperfections in the manufacturing process^18^) and the force induced deflection of the cantilever tip. Moving the cantilever from the x-y position where the approach on the dish surface was made to the position above the cell introduced height shifts of up to ≈ 0.3 *µ*m.

### Transmitosis assay

For transmitosis assays H2B-eGFP and mCherry-CAAX expressing HeLa cell grown in tissue culture flasks were detached by the addition of 1X 0.05% trypsin-EDTA (Invitrogen). The trypsin was deactivated by the addition of a tenfold volume of DMEM with 4 mM Na_2_CO_3_ and 10% fetal bovine serum buffered with 20 mM HEPES/NaOH pH 7.2. The cells were then added to a PDMS (Sylgard 148, Dow Corning) coated glass bottom Petri dish. The PDMS coating prevented cells from adhering to the dish. In the microscope, prophase cells were identified based on H2B-eGFP images and confined to a height of about ≈12 *µ*m. Confocal stacks (1.5 *µ*m z-interval) of mCherry-CAAX were recorded at 5 minute intervals until the cell completed mitosis.

## Author Contributions

E.F.F. and J.H. performed the research and analysed the data. E.F.F., A.A.H., F.J., D.J.M. and J.H. designed the research and wrote the main manuscript.

## Supplementary Material

Supplementary Information Supplementary Information

## Figures and Tables

**Table 1:** Relative changes of geometrical and mechanical cell parameters due to photo-inactivation of blebbistatin. *H* denotes the mean curvature of the fit cell shape. Errors are the (relative) standard error of the mean (N = 13)

*F_AFM_*	Δ*P*	*γ*	*r_max_*	*h_fit_*	*H*
52 ± 12%	63 ± 16%	66 ± 18%	−1 ± 0.3%	2 ± 2%	−1 ± 1%

## References

[b1] Strangeways T. S. P. Observations on the changes seen in living cells during growth and division. P Roy Soc Lond B Bio 94, 137–141 (1922).

[b2] McConnell C. H. Mitosis in hydra. mitosis in the ectodermal epithelio-muscular cells of hydra. Biol Bull 64, 86–95 (1933).

[b3] Sauer F. C. Mitosis in the neural tube. J Comp Neurol 62, 377405 (1935).

[b4] Harris A. Location of cellular adhesions to solid substrata. Dev Biol 35, 97–114 (1973). 4787750 10.1016/0012-1606(73)90009-2

[b5] Luxenburg C., Amalia Pasolli H., Williams S. E. & Fuchs E. Developmental roles for Srf, cortical cytoskeleton and cell shape in epidermal spindle orientation. Nat Cell Biol 13, 203–214 (2011). 21336301 10.1038/ncb2163 PMC3278337

[b6] Morin X. & Bellaïche Y. Mitotic spindle orientation in asymmetric and symmetric cell divisions during animal development. Dev Cell 21, 102–119 (2011). 21763612 10.1016/j.devcel.2011.06.012

[b7] Lancaster O. M. *et al.* Mitotic rounding alters cell geometry to ensure efficient bipolar spindle formation. Dev Cell 25, 270–283 (2013). 23623611 10.1016/j.devcel.2013.03.014

[b8] Théry M., Jiménez-Dalmaroni A., Racine V., Bornens M. & Jülicher F. Experimental and theoretical study of mitotic spindle orientation. Nature 447, 493–496 (2007). 17495931 10.1038/nature05786

[b9] Salbreux G., Charras G. & Paluch E. Actin cortex mechanics and cellular morphogenesis. Trend Cell Biol 22, 536–545 (2012). 10.1016/j.tcb.2012.07.001 22871642

[b10] Lecuit T. & Lenne P.-F. Cell surface mechanics and the control of cell shape, tissue patterns and morphogenesis. Nat Rev Mol Cell Biol 8, 633–644 (2007). 17643125 10.1038/nrm2222

[b11] Clark A. G. & Paluch E. Mechanics and regulation of cell shape during the cell cycle. Results Probl Cell D, 53, 31–73 (2011). 10.1007/978-3-642-19065-0_3 21630140

[b12] Nurse P. Regulation of the eukaryotic cell cycle. Eur J Cancer 33, 1002–1004 (1997). 9376179 10.1016/s0959-8049(97)00091-9

[b13] Cramer L. P. & Mitchison T. J. Investigation of the mechanism of retraction of the cell margin and rearward flow of nodules during mitotic cell rounding. Mol Biol Cell 8, 109–119 (1997). 9017599 10.1091/mbc.8.1.109 PMC276063

[b14] Maddox A. S. & Burridge K. RhoA is required for cortical retraction and rigidity during mitotic cell rounding. J Cell Biol 160, 255–265 (2003). 12538643 10.1083/jcb.200207130 PMC2172639

[b15] Matthews H. K. *et al.* Changes in Ect2 localization couple actomyosin-dependent cell shape changes to mitotic progression. Dev Cell 23, 371–383 (2012). 22898780 10.1016/j.devcel.2012.06.003 PMC3763371

[b16] Usui N. & Yoneda M. Ultrastructural basis of the tension increase in sea-urchin eggs prior to cytokinesis. Dev Growth Differ 24, 453465 (1982). 10.1111/j.1440-169X.1982.00453.x 37280882

[b17] Stewart M. P. *et al.* Hydrostatic pressure and the actomyosin cortex drive mitotic cell rounding. Nature 469, 226–230 (2011). 21196934 10.1038/nature09642

[b18] Stewart M. P. *et al.* Wedged AFM-cantilevers for parallel plate cell mechanics. Methods 59, 186–194 (2013). 23473778 10.1016/j.ymeth.2013.02.015

[b19] Lim C., Zhou E. & Quek S. Mechanical models for living cells – a review. J Biomech 39, 195–216 (2006). 16321622 10.1016/j.jbiomech.2004.12.008

[b20] Yeung A. & Evans E. Cortical shell-liquid core model for passive flow of liquid-like spherical cells into micropipets. Biophys J 56, 139–149 (1989). 2752083 10.1016/S0006-3495(89)82659-1 PMC1280459

[b21] Yoneda M. Tension at the surface of sea-urchin egg: A critical examination of cole's experiment. J Exp Biol 41, 893–906 (1964). 14239916 10.1242/jeb.41.4.893

[b22] Yoneda M. The compression method for determining the surface force. Method Cell Biol 27, 421–434 (1986). 10.1016/s0091-679x(08)60362-3 3517588

[b23] Skoufias D. A. *et al.* S-trityl-l-cysteine is a reversible, tight binding inhibitor of the human kinesin eg5 that specifically blocks mitotic progression. J Biol Chem 281, 17559–17569 (2006). 16507573 10.1074/jbc.M511735200

[b24] Straight A. F. *et al.* Dissecting temporal and spatial control of cytokinesis with a myosin II inhibitor. Science 299, 1743–1747 (2003). 12637748 10.1126/science.1081412

[b25] Sakamoto T., Limouze J., Combs C. A., Straight A. F. & Sellers J. R. Blebbistatin, a myosin II inhibitor, is photoinactivated by blue light. Biochemistry 44, 584–588 (2005). 15641783 10.1021/bi0483357

[b26] Koenderink G. H. *et al.* An active biopolymer network controlled by molecular motors. PNAS 106, 15192–15197 (2009). 19667200 10.1073/pnas.0903974106 PMC2741227

[b27] Tinevez J.-Y. *et al.* Role of cortical tension in bleb growth. PNAS 106, 18581–18586 (2009). 19846787 10.1073/pnas.0903353106 PMC2765453

[b28] Clark A. G., Dierkes K. & Paluch E. K. Monitoring actin cortex thickness in live cells. Biophys J 105, 570–580 (2013). 23931305 10.1016/j.bpj.2013.05.057 PMC3736691

[b29] Cole K. S. Surface forces of the arbacia egg. J Cell Compar Physl 1, 19 (1932).

[b30] Wang L., Hukin D., Pritchard J. & Thomas C. Comparison of plant cell turgor pressure measurement by pressure probe and micromanipulation. Biotechnol Lett 28, 1147–1150 (2006). 16819586 10.1007/s10529-006-9075-x

[b31] Fernández P. & Ott A. Single cell mechanics: stress stiffening and kinematic hardening. Phys Rev Lett 100, 238102 (2008). 18643547 10.1103/PhysRevLett.100.238102

[b32] Schäfer E., Kliesch T.-T. & Janshoff A. Mechanical properties of giant liposomes compressed between two parallel plates: Impact of artificial actin shells. Langmuir 29, 10463–10474 (2013). 23869855 10.1021/la401969t

[b33] Desprat N., Richert A., Simeon J. & Asnacios A. Creep function of a single living cell. Biophys J 88, 2224–2233 (2005). 15596508 10.1529/biophysj.104.050278 PMC1305272

[b34] Morris C. E. & Homann U. Cell surface area regulation and membrane tension. J Membrane Biol 179, 79–102 (2001). 11220366 10.1007/s002320010040

[b35] Evans E. & Kukan B. Passive material behavior of granulocytes based on large deformation and recovery after deformation tests. Blood 64, 1028–1035 (1984). 6487804

[b36] Hochmuth R. M. Micropipette aspiration of living cells. J Biomech 33, 15–22 (2000). 10609514 10.1016/s0021-9290(99)00175-x

[b37] Effler J. C. *et al.* Mitosis-specific mechanosensing and contractile-protein redistribution control cell shape. Curr Biol 16, 1962–1967 (2006). 17027494 10.1016/j.cub.2006.08.027 PMC2474462

[b38] Charras G. T., Coughlin M., Mitchison T. J. & Mahadevan L. Life and times of a cellular bleb. Biophys J 94, 1836–1853 (2008). 17921219 10.1529/biophysj.107.113605 PMC2242777

[b39] Mohr H. & Schopfer P. Plant Physiology, (Springer, 1995). 10.1104/pp.49.1.8 PMC365891 16657900

[b40] Farinas J., Kneen M., Moore M. & Verkman A. S. Plasma membrane water permeability of cultured cells and epithelia measured by light microscopy with spatial filtering. J Gen Physiol 110, 283–296 (1997). 9276754 10.1085/jgp.110.3.283 PMC2229369

[b41] Numata T., Shimizu T. & Okada Y. TRPM7 is a stretch- and swelling-activated cation channel involved in volume regulation in human epithelial cells. Am J Physiol-Cell Ph 292, C460–C467 (2007). 10.1152/ajpcell.00367.2006 16943238

[b42] Boucrot E. & Kirchhausen T. Mammalian cells change volume during mitosis. PLoS ONE 3, e1477 (2008). 18213385 10.1371/journal.pone.0001477 PMC2198941

[b43] Gavet O. & Pines J. Progressive activation of CyclinB1-Cdk1 coordinates entry to mitosis. Dev Cell 18, 533–543 (2010). 20412769 10.1016/j.devcel.2010.02.013 PMC3325599

[b44] Israelachvili J. N. Intermolecular and surface forces, (Academic Press, Amsterdam, 2011).

[b45] Evans E. Equilibrium wetting of surfaces by membrane-covered vesicles. Adv Colloid Interfac 39, 103–128 (1992). 10.1016/0001-8686(92)80057-5 1590974

