# Supplementary material for: Quantification of surface tension and internal pressure generated by single mitotic cells
Source: Sci Rep. 2014 Aug 29;4:6213. doi: 10.1038/srep06213 (PMC4148660; doi:10.1038/srep06213)
Supplement: Supplementary Information [file srep06213-s1.pdf]

Supplementary Material
Quantification of surface tension and internal pressure generated by single mitotic cells

Elisabeth Fischer-Friedrich[1,2], Anthony A. Hyman[2], Frank Jülicher[1], Daniel J. Müller[3], and Jonne Helenius[*3]

[1]Max Planck Institute for the Physics of Complex Systems, Nöthnitzer Strasse 38, 01187 Dresden, Germany
[2]Max Planck Institute of Molecular Cell Biology and Genetics, Pfotenhauerstr. 108, 01307 Dresden, Germany
[3]D-BSSE, Eidgenössische Technische Hochschule Zürich, Mattenstr. 26, 4058 Basel, Switzerland

July 22, 2014

1 Force balance at the cell boundary and Laplace's law

A simple physical model of a cell is that of a pressurized liquid sack bound by a cortical shell in which contractile in-plane tensions are present. In this model, the tensile stresses in the shell lead to an inward force F_{in} on an area element of the shell, while the hydrostatic pressure excess of the fluid inside the shell pushes the area element outwards, creating an outward force F_{out}. Laplace's law states the condition of force balance, i.e. the condition of $F_{in} = F_{out}$ (Fig. S1). As a liquid can be essentially regarded as incompressible, i.e. it has an extremely large elastic bulk modulus, even a very small compression or expansion of the liquid in the cell leads to substantial change of hydrostatic pressure. Therefore, if the mechanical tension in the cortical shell and F_{in} increases, there is a very small compression of the bulk fluid leading to a sudden increase of internal hydrostatic pressure as fluid molecules are forced a little bit more together. As a consequence, the force balance at the cell boundary is realized almost instantly.

[*]Corresponding author. Tel: +41 61 3873330; E-mail address: jonne.helenius@bsse.ethz.ch

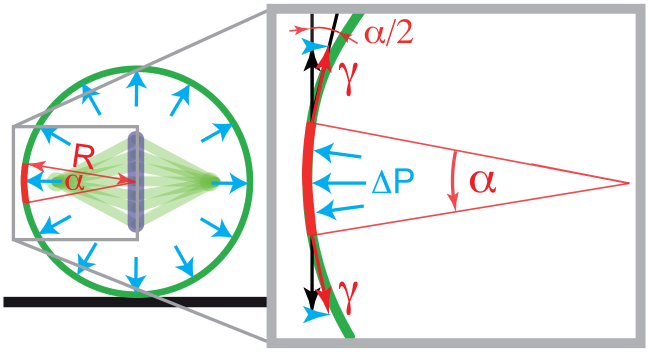

Figure S1: Sketch of force balance at an area element of a spherical cell. The cell has a radius R and a uniform cortical tension γ. The area element (red segment) is square-shaped with side length $ds = R\alpha$, where α is considered to be very small. Therefore, the outward force F_{out} amounts to $ds^2 \Delta P$. The inward force on one edge of the area element is $\gamma \sin(\alpha/2) ds$. As there are four edges in total, the total inward force F_{in} is $\approx 4\gamma \sin(\alpha/2) ds \approx 2\gamma\alpha ds$. Force balance requires then $R^2\alpha^2\Delta P = 2\gamma\alpha^2 R$, which can be simplified to $\Delta P = \frac{2\gamma}{R}$. This is the well-known form of Laplace's law for a sphere.

In the case of a cell, the boundary of the pressurized liquid sack is water-permeable. This might appear contradictory to the fact that the cell maintains an internal hydrostatic pressure excess. If one imagines a water-balloon whose skin is made water-permeable by piercing holes into it, then the balloon will leak water and shrink until the tension in the skin has vanished and the hydrostatic pressure excess in the balloon is zero. However, the same scenario does not apply to a cell; While the cell membrane is permeable for water, it is much less permeable for ions and other solutes within the cytoplasm. Therefore, if the cell leaks water due to an increase in cortical tension, the concentration of solutes in the cytoplasm will increase. This concentration increase will stop water from further leaving the cell, when osmotic pressure excess and hydrostatic pressure excess in the cell are equal. This balance of osmotic and hydrostatic pressure excess is not realized immediately but depends on the speed of water fluxes across the cell membrane.

2 Theoretically predicted cell shapes and fitting to measured cell shapes

Consider a cell confined between parallel plates. If the contact angle of the cell surface with the plates vanishes, a mathematical derivation (1) shows that the vertical cross-section of the free cell surface is described by the curve

$$\pm z(r_{max}, r_{min}, r) = -\frac{r_{min}^2}{r_{max}} F(\Phi, k) + r_{max} E(\Phi, k), \quad r_{min} \leq r \leq r_{max} \quad \text{(S1)}$$

where Φ and k are placeholders defined by the equations

$$\sin(\Phi) = (r_{max}^2 - r^2)^{\frac{1}{2}} (k r_{max})^{-1},\ k = (r_{max}^4 - r_{min}^4)^{\frac{1}{2}} / r_{max}^2 \quad \text{(S2)}$$

and r_{max} is the radius of the cell at the equator while r_{min} is the radius of the top (and bottom) cross section (see Fig. S2A). $F(\Phi, k)$ and $E(\Phi, k)$ are elliptic integrals of the first and second kind, respectively. By revolution of this curve around the z-axis, one obtains a 3-D surface. This surface is parameterized by its minimal and maximal cross sectional radii (r_{min}, r_{max}). A shape having a non-vanishing contact angle φ at the top plate (and analogously at the bottom plate) is realized by limiting the above curve to a smaller interval $r \in [r_c, r_{max}]$ with $r_c > r_{min}$ in the positive z-range such that $\theta(z(r_c)) = \varphi$ (see Fig. S2B,D), where $\theta(z)$ is the tangential angle with $\tan(\theta(z)) = -dz/dr$. A theoretical cell shape is parameterized by a center point (x_c, y_c, z_c) and the shape's two radii, r_{min} and r_{max}. The theoretical shapes are considered to extend up to zero tangential angle - otherwise contact angle would be an additional shape parameter. The height of the fitted theoretical shape h_{fit} is therefore expected to be higher or equal to the cell height h_{AFM} (within measurement errors). The excess of h_{fit} over cell height is a measure for the magnitude of the contact angle of the cell (see Fig. S2C).

For our data analysis, we fit theoretically predicted shapes to experimentally determined cell shapes that are given by a set of N points in 3-D space $\{x_i^{cell}, y_i^{cell}, z_i^{cell}\}_{i=1}^{N}$. Our algorithm fits a shape by a least squares fit. The degree of agreement between the two shapes is assessed according to the distance of measured surface points from the calculated rotationally symmetric shape in 3-D. To calculate the residual, measured 3-D surface data points are first rotated around the z-axis into the x-z-plane, such that the coordinates in the plane are $(x_i^{cell,2D}, z_i^{cell,2D}) = (\sqrt{(x_i^{cell} - x_c)^2 + (y_i^{cell} - y_c)^2}, z_i^{cell} - z_c)$. The residual is the distance of the data point to the vertical cross section of the theoretical cell shape

$$res_i = \sqrt{(x_i^{cell,2D} - r_i^{fit})^2 + (z_i^{cell,2D} - z_i^{fit})^2},\ i = 1 \dots N. \quad \text{(S3)}$$

where (r_i^{fit}, z_i^{fit}) is the closest point on the curve given by Eq. S1.

The error of determined fit parameters was calculated as commonly established for least square fitting by assuming that the errors are normally distributed (3). The fit shape is parameterized by $\boldsymbol{\beta} = (x_c, y_c, z_c, r_{max}, r_{min})$ and defines a grid of fit space points. The error of the jth fit parameter is calculated as

$$\sqrt{\frac{||res||^2}{n-5}(X^T X)^{-1}_{jj}},$$

where X is the Jacobian matrix

$$X_{ij} = \frac{\partial res_i}{\partial \beta_j}.$$

We estimated the overall error of measured, geometrical cell parameters in the following way: the error of the cross sectional radii r_{max} and r_{min} was deemed to be either the optical resolution (250 nm) or the mean distance between the fit theoretical shape and the measured cell shape, $< d >$, whichever was greater. To a good approximation, the vertical cross-section of the free cell surface is a semi-circle such that cell height can be approximated by $h_{fit} \approx 2(r_{max} - r_{min})$. This is used to estimate the error of the fit height as $\Delta h_{fit} \approx 2(\Delta r_{max} + \Delta r_{min}) \approx 4 \max(< d >, r)$. Therefore, for a value of $< d >= 250$ nm, the estimated error of the fit height is $\approx 1\,\mu$m.

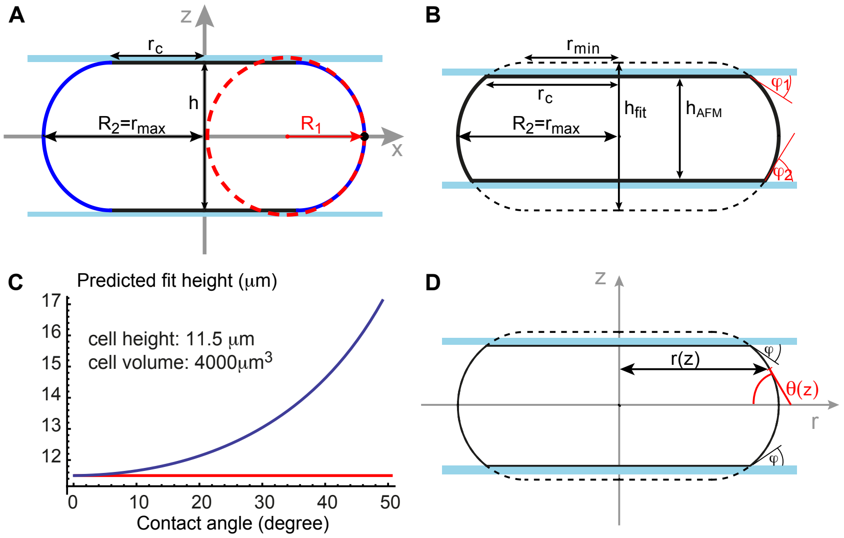

Figure S2: The effect of cell adhesion on the cell shape as predicted by theory. (A) Depiction of the theoretical shape of a cell that has contact angles equal to zero (vanishing adhesion), a maximal radius of $r_{max} = 12\,\mu$m, a contact radius of $r_c = 7\,\mu$m and a height of $11.08\,\mu$m (This height results from setting the two radii.). The shape of the vertical cross-section of the free cell surface (blue solid curve) is given by Eq. S1. The radii of principal curvatures of a point at the cell equator are R_1 and $R_2 = r_{max}$. (B) Depiction of the theoretical shape of a cell with finite contact angles due to adhesion at top and bottom interfaces. If adhesion strength at top and bottom differs, contact angles φ_1 and φ_2 as well as contact areas at top and bottom are not equal. The fitting of a theoretically predicted shape associates a shape represented by the dashed profile. The height of the theoretical shape, h_{fit}, is larger than the actual height, h_{AFM}, of the adhering cell. (C) Fit height h_{fit} (blue) and actual cell height h_{AFM} (red) of cell shapes with non-vanishing contact angle (equal at top and bottom) as predicted by theory. The actual cell height, $h_{AFM} = 11.5\,\mu$m, and the cell volume, $4000\,\mu\text{m}^3$, are constant. (D) The function $r(z)$ defines the cross-sectional radius at height coordinate z. $\theta(z)$ is the angle between the associated tangent and the x-axis.

Marker	r_{max} (μm± s.e.)	r_{min} (μm± s.e.)	h_{fit} (μm± s.e.)	h_{AFM} (μm)	$< d >$ (μm)
Cell 1, Lifeact	11.26±0.01	5.72±0.03	12.41±0.07	11.8±0.3	0.19
Cell 1, Myosin II	11.21±0.01	5.66±0.03	12.44±0.07	11.8±0.3	0.17
Cell 2, Lifeact	10.65±0.01	4.24±0.03	13.36±0.08	13.8±0.3	0.26
Cell 2, Myosin II	10.65±0.01	4.42±0.04	14.1±0.09	13.8±0.3	0.26
Cell 3, Lifeact	9.99±0.01	4.9±0.03	11.43±0.09	11.6±0.3	0.21
Cell 3, Myosin II	9.86±0.01	4.86±0.02	11.23±0.06	11.6±0.3	0.15
Cell 4, Lifeact	11.2±0.01	5.6±0.04	12.56±0.1	11.6±0.5	0.25
Cell 4, Myosin II	11.17±0.01	5.61±0.04	12.47±0.1	11.6±0.3	0.26
Cell 5, CAAX	10.3±0.01	4.55±0.03	13.0±0.07	11.6±0.3	0.2
Cell 6, CAAX	10.83±0.01	5.3±0.02	12.4±0.06	11.9±0.3	0.16
Cell 7, CAAX	10.6±0.01	5.17±0.03	12.2±0.08	11.7±0.3	0.23

Table S1: Theoretically predicted shapes were fit to confocal z-stacks ($\Delta z = 0.5\,\mu$m) of confined metaphase cells that had fluorescently labeled cell boundaries. The first two lines are associated to the fits presented in Fig. 2, main text. Fit parameters were the center of the shape (not given) and the top and middle cross sectional radii, r_{min} and r_{max}. The error of the AFM height h_{AFM} is estimated based on the roughness of the cantilever wedge and the mean shift in cantilever height that occurs while positioning the cantilever onto the cell (see Methods). The height of the fit shape h_{fit} is calculated using Eq. S1. The average distance $< d >$ between measured surface points and the fit surface rates the quality of the fit.

Interphase cell	r_{max} (μm± s.e.)	r_{min} (μm± s.e.)	h_{fit} (μm± s.e.)	h_{AFM} (μm± s.e.)	$< d >$ (μm)
Cell 1	10.13 ± 0.01	4.5 ± 0.03	12.7 ± 0.09	12.5 ± 0.5	0.21
Cell 2	11.87 ± 0.01	5.99 ± 0.03	13.18 ± 0.07	12.5 ± 0.5	0.21
Cell 3	11.15 ± 0.01	6.2 ± 0.03	11.02 ± 0.07	11 ± 0.5	0.2
Cell 4	9.83 ± 0.01	4.16 ± 0.03	12.8 ± 0.06	12 ± 0.5	0.19
Cell 5	9.83 ± 0.01	4.22 ± 0.03	12.67 ± 0.07	12.5 ± 0.5	0.21
Cell 6	10.22 ± 0.01	4.91 ± 0.03	11.94 ± 0.07	12 ± 0.5	0.24
Cell 7	10.18 ± 0.01	4.37 ± 0.03	13.12 ± 0.07	12.5 ± 0.5	0.19
Cell 8	10.27 ± 0.02	4.94 ± 0.04	11.98 ± 0.1	11 ± 0.5	0.29
Cell 9	9.71 ± 0.01	4.12 ± 0.04	12.63 ± 0.09	12 ± 0.5	0.24
Cell 10	9.84 ± 0.01	3.76 ± 0.03	13.76 ± 0.07	13 ± 0.5	0.25
Cell 11	9.09 ± 0.01	3.53 ± 0.02	12.58 ± 0.06	12.5 ± 0.5	0.24

Table S2: Theoretically predicted shapes were fit to confocal z-stacks ($\Delta z = 0.5\,\mu$m) of confined interphase cells with fluorescently labeled cell boundaries. Fit parameters were the center of the shape (not given) and the top and middle cross sectional radii, r_{min} and r_{max}. $< d >$ is the mean distance between the fit shape and the measured cell surface grid. The height of the fit shape h_{fit} is calculated using Eq. S1.

3 Adhesion and cellular contact angle

If cell adhesion to the confining plates is non-negligible, the force balance equation at the plates reads (2)

$$F_{AFM} = \Delta P \pi r_c^2 - 2\pi r_c \gamma \sin(\varphi). \tag{S4}$$

Due to the negative term $2\pi r_c \gamma \sin(\varphi)$, the measured force F_{AFM} can become negative especially if contact areas of the cell with the confining plates are small ($r_c < 2\gamma \sin(\varphi)/\Delta P$, Figure S3A). Then, the cell pulls the cantilever downward. The magnitude of the maximal downward force depends on the strength of cell adhesion and on cell surface tension (Fig. S3B). To probe this experimentally, we performed experiments where we first confined mitotic cells and then slowly lifted the cantilever ($v \leq 0.02 \mu\mathrm{m\,s}^{-1}$) until it detached from the cell and the force stayed constantly zero. We checked that the movement of the cantilever is slow enough to change cell shape adiabatically i.e. without dissipative contributions to the measured AFM force. We find that the cells exert little or no downward forces ($F_{AFM} \geq -1$ nN) on the cantilever before the cantilever detaches (see Fig. S3A). Comparing the measured forces to theoretical predictions, we conclude that the contact angle of a metaphase cell is $\leq 10°$ (see Fig. S3).

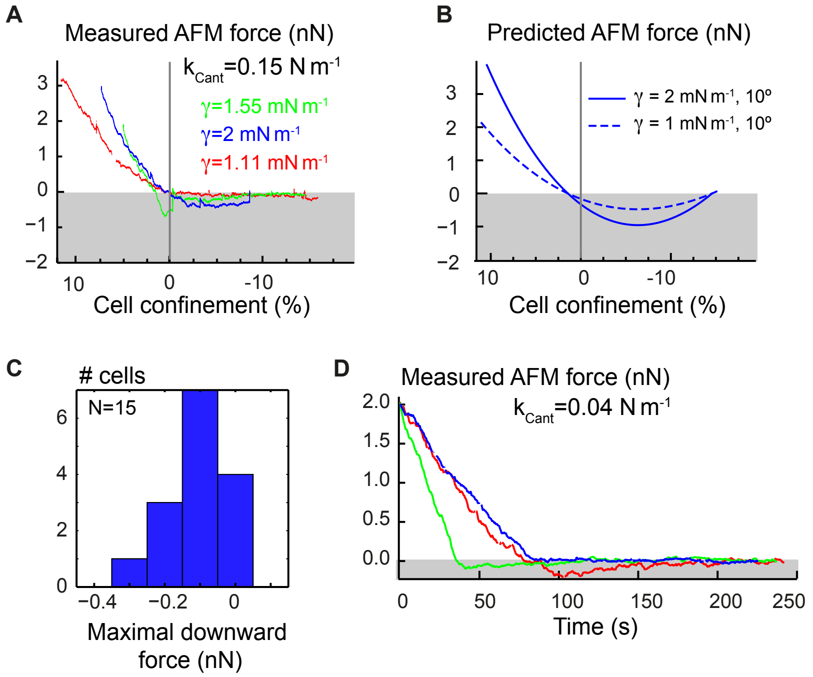

Figure S3: Quantifying the effect of adhesion on cell shape. (A) AFM forces versus degree of cell confinement $1 - h_{AFM}/(2R_0)$ for three confined cells. The force was recorded while the cantilever was raised from a height of 18 μm until it detached from the cell at a speed of $\leq 0.02\,\mu\mathrm{m\,s}^{-1}$ (cantilever stiffness $k = 0.15\,\mathrm{N\,m}^{-1}$). A cell can exert a downward force on the AFM cantilever if it adheres to both substrate and cantilever. (B) Predicted forces for cells with surface tensions $\gamma = 1\,\mathrm{mN\,m}^{-1}$ (dashed curves) or $\gamma = 2\,\mathrm{mN\,m}^{-1}$ (solid curves) and adhesion induced contact angles of $\varphi = 10°$ (blue curves). In the calculations, the volume of the cell was kept at 4000 $\mu\mathrm{m}^3$. For $\varphi = 10°$, the maximum downward force value reached is ≈ -1 nN for the case of $\gamma = 2\,\mathrm{mN\,m}^{-1}$. For calculations in B, we approximated the vertical cross-section of the free cell surface by a segment of a circle. This leads to the equations $R_1 = h/2/\cos(\varphi)$ and $r_c = r_{max} - R_1(1 - \sin(\varphi))$, where R_1 is one of the radii of principle curvatures at the cell equator. (C,D) Measurements as presented in A were repeated with a softer cantilever ($k = 0.04\,\mathrm{N\,m}^{-1}$). (C) Histogram of maximal downward forces reached during the slow cantilever lifting applied to 15 cells. (D) Exemplary force evolution over time for three cells.

4 Dependence of pressure and surface tension on the degree of uniaxial cell confinement

We tested if hydrostatic pressure and surface tension measurements depend on the degree of confinement of the cell (Fig. S4). Mitotic cells were STC arrested and assayed at different degrees of cellular confinement. The degree of cell confinement was defined as $1 - h_{AFM}/(2R_0)$ where R_0 is the radius of the cell in spherical shape and h_{AFM} is the height of the AFM cantilever above the substrate. We found that on average tension values are largely independent of the degree of confinement ($\leq 15\%$ change, Fig. S4D). Pressure values increased slightly with the degree of confinement (Fig. S4C). This is an expected trend as the mean curvature of the cell surface increases with the degree of confinement. Errors in tension and pressure values decrease with higher degrees of confinement (Fig. S4A,B).

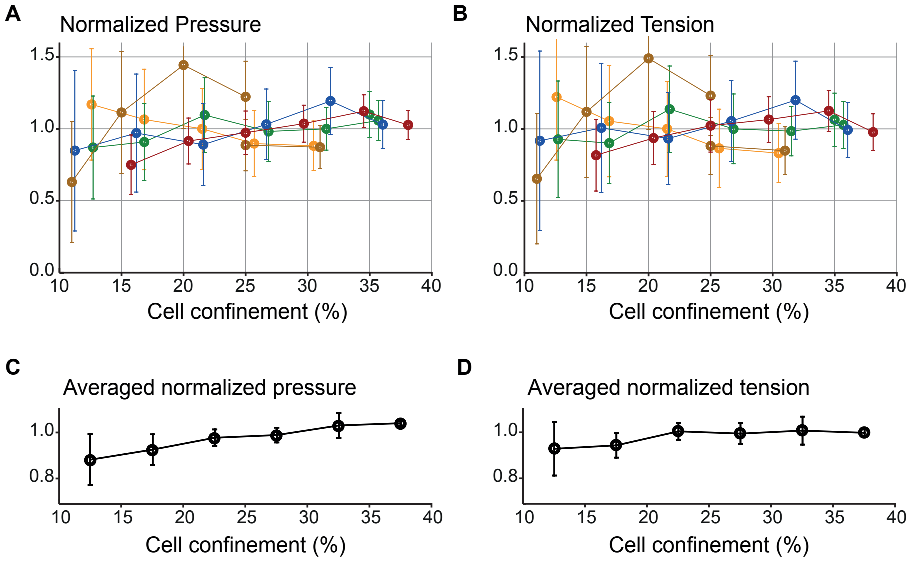

Figure S4: Normalized hydrostatic pressure difference and surface tension of five mitotic, STC arrested cells at different degrees of confinement. (A) Normalized pressures of individual cells (pressures of each cell were divided by their median) at different degrees of cell confinement. For each cell, the cell height was decreased by moving the cantilever downward in $1\,\mu$m increments until large non-retracting blebs were observed. Error bars were obtained by error propagation, assuming a force error of 2 nN and an error $\Delta r_{min} = \Delta r_{max} =< d >$, where $< d >$ is the mean distance of measured shape points from the fit theoretical shape. (B) Normalized surface tension with respect to the degree of cell confinement. Error bars are calculated as in A. (C) Average normalized pressure within bins of 10% height change. (D) Average normalized surface tension within bins of 10% height change. Error bars in C and D denote standard error of the mean.

5 Determining cell shape without recording confocal z-stacks

The theoretical shape of a confined cell with vanishing contact angles is entirely determined by the cell's height h_{AFM} and its radius at the equator r_{max}. These two parameters can be measured using cantilever height and a single fluorescence[1] or DIC micrograph of the equatorial plane of the cell. If cellular adhesion is negligible, the radius of the cell's contact area r_c can be calculated from h_{AFM} and r_{max} (in this case $r_c = r_{min}$.). This derivation of r_c, in turn, allows pressure and cell surface tension to be calculated (Equations 7, 8, main text). Compared to the recording of confocal z-stacks, this single image shape finding method (SIS) is quicker and causes less photo-damage to the cell.

If the actual contact angle adopted by the cell is not zero, the SIS yields smaller surface tensions and the inaccuracy grows with the contact angle (see Fig. S5A). However, the deviation from the actual tension value decreases with increasing degree of cellular confinement. In Section 3, we estimated the adhesive energy and the contact angle and found that contact angles were $\leq 10^\circ$ and probably smaller for most cells examined using our measurement conditions. Fig. S5A compares the calculated cell surface tension obtained under the (false) assumption of vanishing contact angle. For the contact angle values expected, the error in tension due to the simplifying assumption of vanishing contact angle is small ($\leq 10\%$) for typically used degrees of cell confinement.

In Table S3, we give values of the minimal cross-sectional cell radii r_{min} derived from the full 3-D analysis of cell shapes and from the simpler SIS, for the cells presented in Table S1. The agreement of calculated minimal cell radii is good, in particular if AFM height and fit height agree. On average, contact area estimates are 10% bigger if derived with the SIS. For the SIS, we used Eq. S1 to calculate r_c from the AFM height and r_{max}. Since r_c follows only implicitly from this relation, it is helpful to use the heuristic approximate equation

$$r_c \approx (r_{max} - \frac{h_{AFM}}{2}) + \frac{2}{3}\left(\frac{\sqrt{2r_{max}^3 - h_{AFM}r_{max}^2}}{\sqrt{h_{AFM} + 2r_{max}}} - (r_{max} - \frac{h_{AFM}}{2})\right). \quad \text{(S5)}$$

The contact radius can also be estimated in an even simpler way by approximating the vertical profile of the free cell surface as semicircle giving

$$r_c \approx r_{max} - h_{AFM}/2. \quad \text{(S6)}$$

Figure S5B compares the contact radii calculated by the approximate formulae S5 and S6 with the exact numerical calculation for a cell with a volume of $4500\,\mu\text{m}^3$ in dependence of cantilever height. The semicircle approximation introduces substantial errors at small degrees of cell confinement. For

[1] Acquired with a wide-field or confocal microscope.

the cell shape analyses presented in Table S3, contact area estimates are on average 15% smaller if the semicircle approximation is used as compared to the full 3-D analysis.

Marker	r_{max} (μm)	h_{AFM} (μm)	r_{min} (μm)	r_c^{sim} (μm)	$(r_c^{\text{sim}}/r_{min})^2$	r_c^{SC} (μm)	$(r_c^{\text{SC}}/r_{min})^2$
Cell 1, Lifeact	11.26	11.8	5.72	5.98	1.09	5.36	0.88
Cell 1, Myosin II	11.21	11.8	5.66	5.93	1.1	5.31	0.88
Cell 2, Lifeact	10.65	13.8	4.24	4.53	1.14	3.75	0.78
Cell 2, Myosin II	10.65	13.8	4.42	4.53	1.05	3.75	0.72
Cell 3, Lifeact	9.99	11.6	4.9	4.83	0.97	4.19	0.73
Cell 3, Myosin II	9.86	11.6	4.86	4.7	0.94	4.06	0.7
Cell 4, Lifeact	11.2	11.6	5.6	6.01	1.15	5.4	0.93
Cell 4, Myosin II	11.17	11.6	5.61	5.98	1.14	5.37	0.92
Cell 5, CAAX	10.3	11.6	4.55	5.13	1.27	4.5	0.98
Cell 6, CAAX	10.83	11.9	5.3	5.52	1.08	4.88	0.85
Cell 7, CAAX	10.6	11.7	5.17	5.38	1.08	4.75	0.84

Table S3: Comparison of three methods to determine contact radii and contact areas of confined cells. The cells presented are the same as in Table S1. In the above table, r_{min} is the contact radius derived by fitting of a theoretical cell shape to surface grid points from a confocal z-stack ($\Delta z = 0.5\,\mu$m). r_c^{sim} is the contact radius derived from the single image shape finding method (SIS) using the equatorial radius r_{max} and the actual cell height h_{AFM} with the assumption that contact angles are zero. r_c^{SC} is the contact radius as derived from the SIS with the additional approximate assumption that the vertical profile of the free cell surface is a semicircle such that $r_c^{\text{SC}} = r_{max} - h_{AFM}/2$.

We conclude that, although the SIS is somewhat less exact than the 3-D shape analysis described in this paper ($\leq$ 10% error, see Fig. S5A), it is considerably less cumbersome and leads to good approximations of the geometry of cells. Thus, it can be applied to find the hydrostatic pressure and surface tension of a confined cell. The semicircle approximation is a further simplification that yields good results at sufficiently large cell confinement (see Fig. S5B).

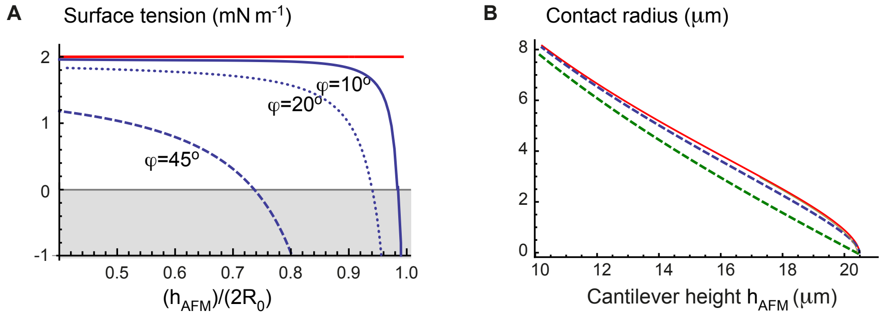

Figure S5: Determining contact radius and cell surface tension with the single image method. (A) Illustration of the error in calculated surface tension due to the assumption of vanishing contact angle. The actual surface tension is $2\,\mathrm{mN\,m^{-1}}$ (red line). The estimated surface tensions (blue lines) are calculated assuming contact angles of $\varphi = 10°$, $20°$ and $45°$ (solid blue, dotted blue and dashed blue lines, respectively). For negative tensions the circle approximation was used, as described in the caption of Fig. S3B. (B) Plot of the contact radius of a theoretical shape with a volume of $4500\,\mu\mathrm{m}^3$ and vanishing contact angle in dependence of the shape's height. Exact solution (red) and solution if formula S5 (dashed blue) or formula S6 (dashed green) is used.

6 Cell edge detection

Images were processed and analyzed using custom code (Igor Pro, Wavemetrics). Each image within a confocal z-stack was processed separately. The cell boundary in each image of a stack is represented by 48 points $\{x_i^{cell}, y_i^{cell}\}_{i=1}^{N=48}$ whose location was found by an edge finding algorithm. Together with the associated z-coordinates, these points constitute a discrete spatial grid that describes the cell shape.

The algorithm for detecting the near circular cell edge of a cell in a fluorescence microscopy image is described in the following. First, the circular path of highest mean pixel intensity is found. This circle is then represented by 48 discrete pixel points $\{x_I^c, y_I^c\}_{I=1}^{N=48}$. Thereafter, the cell boundary is refined using a Viterbi algorithm. The Viterbi algorithm applies in the context of a hidden Markov model. The aim of the algorithm is to map a series of (measured) observables to a series of state variables. In our case, the fluorescence intensity profiles of $N = 48$ lines orthogonal to the circle and passing through one of the discrete points on the circle are the series of observables. The state space associated to each point (x_I^c, y_I^c) on the circle consists of a fixed number N_s of equidistant points in the image that represent a line[2] orthogonal to the circle in point (x_I^c, y_I^c). The states will be referred to by an integer index in the range $1 \ldots N_s$.

We assign a cost for a transition from one state to another in a series of

[2]This line segment is centered on the circle and extends 0.2 circle radii into and beyond the circle. The number of pixels that fit into this segment sets the value of N_s.

states and then find the series of states of length N that has a minimal cost associated with it. Given the cost of a state series with $(I-1)$ elements, the cost of adding the state j as Ith element is defined as

$$s_I(\ldots,k,j) \;\; = \;\; s_{I-1}(\ldots,,k) - \log\left(\mathcal{I}_{I,j}\right) - \log\left(f(0,\sigma^2,|j-k|)\right), \quad \text{(S7)}$$

where k is precursor state in the series. Here, f is a Gaussian function with variance σ that is obtained by fitting a Gaussian function to the fluorescence profile orthogonal to the circle at point (x_1^c, y_1^c). The $\mathcal{I}_{I,j}$ is the averaged intensity in the neighbourhood of point j on intensity line profile I

Not knowing the optimal state series, the algorithm considers the cost of all possible state series and chooses the one with the lowest cost. To find the optimal state series, two arrays A and B are introduced. While, the first row of B stays empty, in A it is filled with normalized intensities

$$A_{0,j} = -\log\left(\mathcal{I}_{0,j}\right). \quad \text{(S8)}$$

After the first row the arrays are filled according to:

$$A_{I,j} \;\; = \;\; \min_k \left(A_{I-1,,k} - \log\left(\mathcal{I}_{I,j}\right) - \log\left(f(0,\sigma^2,|j-k|)\right)\right) \quad \text{(S9)}$$

$$B_{I,j} \;\; = \;\; \arg\min_k \left(A_{I-1,,k} - \log\left(\mathcal{I}_{I,j}\right) - \log\left(f(0,\sigma^2,|j-k|)\right)\right) \quad \text{(S10)}$$

Thus, the entry $A_{I,j}$ stores the minimal cost of a path of length I with final state j.

We now find the series of states that gives the lowest cost in total. Hereto, the entry in the last row of A with the minimal cost is selected, say A_{N,j_N}. This identifies the index of the last state in the optimal series as j_N. Now the corresponding entry is picked in B, namely B_{N,j_N} which gives the index j_{N-1} of the second last state in the optimal state series. In this manner, the optimal series $(j_1,\ldots,,j_I,\ldots,j_N)$ is extracted from array B: $B_{I,j_I} = j_{I-1}$.

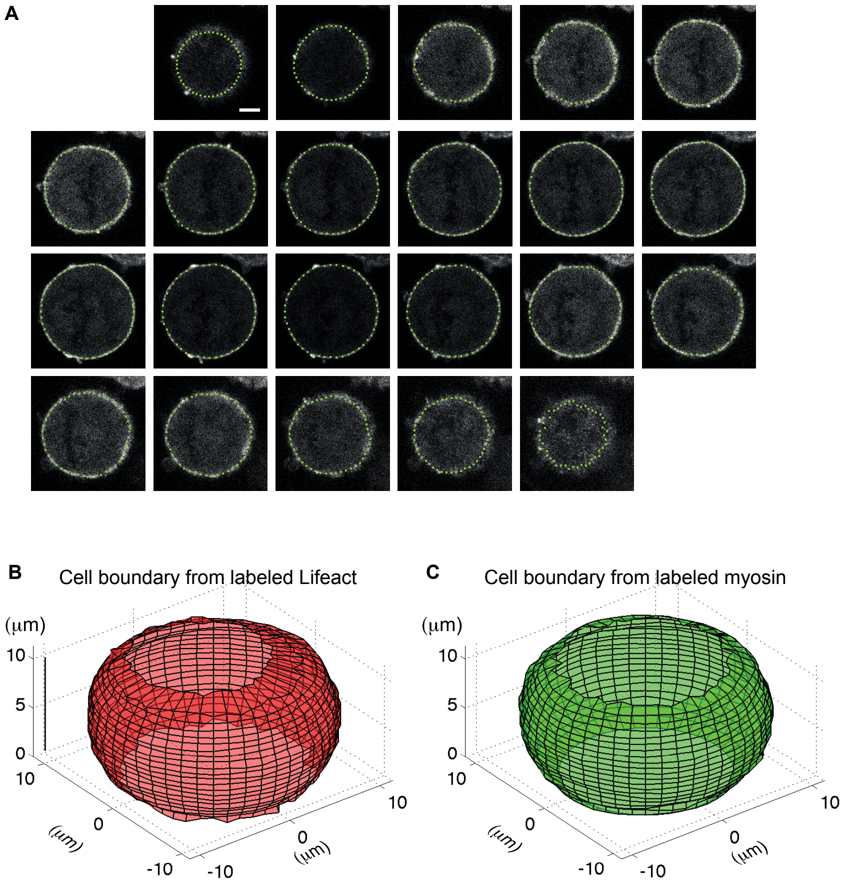

Figure S6: Three-dimensional cell shape. (A) Images in a confocal z-stack acquired form a confined MYH9-eGFP expressing HeLa cell. Green points denote cell surface points found using the edge finding algorithm. Scale bar, 5 μm. (B and C) Depictions of the 3-D grid of the cellular shape obtained from confocal images of fluorescent tagged Lifeact (B) and myosin II (C) of the same cell. The grid in C was obtained from the images in A. The images and grids are the same as used in Fig. 2 of the main text.

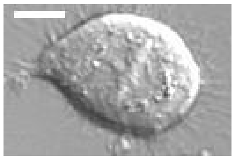

Figure S7: DIC image of a metaphase cell (HeLa-Kyoto, H2B-eGFP, mCherry-CAAX) after incubation with blebbistatin (10 μM) for $\approx$ 90 minutes on an uncoated glass bottom dish. Incomplete cell-rounding is typical of myosin II inhibited, surface-bound HeLa cells and testifies to the significance of actomyosin contraction for mitotic cell-rounding. Scale bar, 10 μm.

7 Calculating cell volume

The volume of a cell was calculated from its 3-D surface grid $\{x_i^{cell}, y_i^{cell}, z_i^{cell}\}_{i=1}^{N}$. We regarded the uppermost and lowermost horizontal cross sections of a grid as the top and bottom boundary of an enclosed volume. As each grid cross section contains the same number of points, triangulating the nearly square-grid surface is done by dividing each surface quadrangle into two triangles. The top and bottom cross sections are triangulated by connecting the surface points at its boundary with the center point of the cross section. The entire enclosed volume is then segmented into tetrahedral volumes defined by connecting all the triangles on the surface with the same point, which lies in the middle of the volume. The sum of tetrahedral volumes is used as the cell volume.

References

[1] Yoneda M (1964) Tension at the surface of sea-urchin egg: A critical examination of Cole's experiment. *J Exp Biol* 41:893–906.

[2] Israelachvili JN (2011) *Intermolecular and surface forces* (Elsevier, Acad. Press, Amsterdam [u.a.]), 3. ed. edition.

[3] Schittkowski, K (2002) *Numerical data fitting in dynamical systems* (Kluwer Academic Publishers)
